# Interactions of *Lactobacilli* with Pathogenic *Streptococcus pyogenes*

**DOI:** 10.1155/2010/289743

**Published:** 2010-05-24

**Authors:** Mark L. Westbroek, Crystal L. Davis, Lena S. Fawson, Travis M. Price

**Affiliations:** Department of Clinical Laboratory Sciences, Ezekiel R. Dumke College of Health Professions, Weber State University, 3905 University Circle, Ogden, UT 84408-3905, USA

## Abstract

*Objective*. To determine whether (1) a decreased concentration of *Lactobacilli* allows *S. pyogenes* to grow; (2) *S. pyogenes* is able to grow in the presence of healthy *Lactobacillus* concentrations; (3) *S. pyogenes* is capable of inhibiting *Lactobacilli*. *Methods*. One hundred fifty patient samples of *S. pyogenes* were mixed with four different concentrations of *L. crispatus* and *L. jensenii*. Colony counts and pH measurements were taken from these concentrations and compared using *t*-tests and ANOVA statistical analyses. *Results*. Statistical tests showed no significant difference between the colony counts of *S. pyogenes* by itself and growth when mixed with *Lactobacilli*, and no significant difference between the colony counts of *S. pyogenes* in the four different concentrations of *Lactobacilli*. *Conclusion*. The statistical data representing the growth of these two organisms suggests that *Lactobacilli* did not inhibit the growth of *S. pyogenes*. Also, *S. pyogenes* did not inhibit the growth of *Lactobacilli*.

## 1. Introduction

*Lactobacillus* bacteria (*Lactobacilli*) are large Gram positive rods that exist as nonpathogenic microbiota. *Lactobacilli* have been extensively studied due to their remarkable ability to inhibit the growth of other organisms through bactericidal activity and by producing lactic acid as a byproduct of metabolism [1, 2]. Lactic acid production, production of bacteriocins, and the production of hydrogen peroxide have led to an abundance of research involving the ability of *Lactobacilli* to inhibit pathogens. *Lactobacillus* species have proven effective at inhibiting the growth of bacterial and fungal pathogens which commonly cause vaginosis. *Lactobacillus* species, specifically *L. crispatus* and *L. jensenii*, are the predominant flora in the vagina, and thus minimize opportunities for infection [3–8]. Several common pathogens that *Lactobacilli *inhibit are: *Candida albicans*, *Escherichia coli* (including *E. coli O157:H7*); and *Neisseria gonorrhoeae *[1, 2, 5–9]. 

Due to their ability to inhibit other organisms, *Lactobacilli* are commonly used for probiotic therapy to enhance intestinal microbiota, as well as to treat vaginosis. The principle of this treatment is to increase the concentration of *Lactobacilli*, which will inhibit pathogens and allow the body's immune system to overcome the infection without the use of antimicrobials [8, 9]. 

*Streptococcus pyogenes*, often referred to as Group A strep, is a Gram positive coccus which tends to group together in chains. *S. pyogenes* causes the infection commonly known as “strep throat” and is the cause of 90% of bacterial pharyngitis cases. It can cause impetigo, erysipelas (cellulitis), toxic shock syndrome, and necrotizing fasciitis (also known as “flesh-eating strep”). Untreated infections may lead to acute glomerulonephritis, scarlet fever, or rheumatic fever. It has many virulence factors that contribute to its pathogenicity, such as lipoteichoic acid, M protein, hyaluronidase, protease, streptokinase, DNase/RNase, C5a peptidase, and Streptolysins O and S. These allow the bacteria to hemolyze blood cells, spread throughout the body, adhere to surfaces, and necrotize tissues [10]. 

*S. pyogenes* can exist in the vagina [4], but it was not previously considered a cause of bacterial vaginosis [11–13]. However, the recorded incidence of bacterial vaginosis caused by *S. pyogenes* has increased during the past two decades [14–16]. Studies regarding this increased prevalence suggest that the pathogen is introduced to the genital area by persons that carry *S. pyogenes* in their respiratory tract either as normal flora or as a pharyngeal infection [11, 12, 17–20]. In response to the increase in occurrence, many laboratories are beginning to make changes to protocols regarding the detection of *S. pyogenes*. Many protocols now include *S. pyogenes* as a potential vaginal pathogen that, in addition to other vaginal pathogens, needs to be identified when present. 

Research indicates that in most cases of bacterial vaginosis, the *Lactobacillus* concentration is notably decreased [9, 20, 21], thus allowing an infection to take place. The decreased concentration of *Lactobacilli *is often due to the use of antimicrobials. This study addresses the following questions.

1. Does a decreased concentration of *Lactobacilli* allow *S. pyogenes* to grow?
2. Is *S. pyogenes* able to grow in the presence of healthy *Lactobacillus* concentrations?
3. Is *S. pyogenes* capable of inhibiting *Lactobacilli*?

Previous studies show that the average healthy vagina has a concentration of about 10^6^ colony-forming units per milliliter (CFU/mL) of *Lactobacilli*. The average healthy vaginal pH is about 3.5–4.8 [3, 4, 15, 22]. Although uncommon, the pH can reach as high as 8.0, depending on which part of the menstrual cycle is occurring. The fluctuation of hormone levels associated with the menstrual cycle affects the concentration of *Lactobacilli*, leading to a fluctuation in pH. Symptomatic cases of vaginosis usually have low concentrations of *Lactobacilli*, accompanied by an increased pH (≥7.0) [1, 4, 5, 23].

## 2. Materials and Methods

In order to minimize variation in *Lactobacillus* species that might be found in clinical specimens, strains of *L. crispatus* and *L. jensenii* were purchased from the American Type Culture Collection (ATCC). *L. crispatus* (ATCC 33197) and *L. jensenii* (ATCC 25258) were mixed in sterile Columbia broth (Fisher Scientific, Pittsburgh, PA) to concentrations of 10^8^, 10^6^, 10^4^, and 10^3^ CFU/mL [23]. The 10^6^ is representative of the average *Lactobacillus* concentration in a healthy female [5]. A higher than average concentration was set up to represent the females who have more *Lactobacillus*, along with two lower concentrations to represent individuals who would be at a higher risk of infection. Columbia broth was chosen because it did not favor the growth of either organism, and it allowed the fluctuations in pH to be measured easily.

The source of bacterial vaginosis caused by *S. pyogenes* is suspected to be the throat, so 150 positive *S. pyogenes* throat screens were donated by Ogden Clinic in Ogden, Utah. Personal identification information of each patient was removed by the clinic before donating the samples, earning this study an exempt status from the Weber State University Institutional Review Board approval. Subcultures were performed in order to isolate and verify the identity of *S. pyogenes*. Research indicates that the average concentration of pathogens which cause bacterial vaginosis is about 10^3^ CFU/mL [16]. However, the concentration of *S. pyogenes* in saliva has not yet been studied. A preliminary experiment was conducted to determine a concentration to use. For this preliminary experiment, concentrations of *S. pyogenes* at 10^2^ and 10^3^ CFU/mL were grown with *Lactobacilli* at 10^6^ CFU/mL. A concentration of 10^3^ CFU/mL was used, based on the colony counts from these concentrations.

A nephelometer, which is an instrument that measures the turbidity of liquid solutions, was used to measure the concentrations of the bacteria in the broth preparations, followed by serial dilutions to achieve the various desired concentrations. Three mL preparations of *S. pyogenes* at 10^3^ CFU/mL were mixed with three mL of each of the four different concentrations of* Lactobacilli*. Each of these four mixtures was then plated on Columbia Sheep Blood Agar (SBA) (Fisher Scientific, Pittsburgh, PA) using calibrated 1.0 *μ*L loops. The broth mixtures and SBA plates were incubated in a carbon dioxide incubator at 37°C for 48 hours. After 48 hours, colonies were counted on the SBA plates, and pH measurements were taken from each broth mixture.Colonies were counted at 48 hours because the *Lactobacillus* colonies were larger and easier to count after 48 hours. There was no difference in colony counts or pH levels between 24 and 48 hours.

To validate the methodology used in this study, the same process was repeated using *Escherichia coli*, *Streptococcus agalactiae* (Group B strep), *Staphylococcus epidermidis*, and *Staphylococcus aureus*, which are known to be inhibited by *Lactobacilli*. Each of these was plated on an SBA plate before mixing with the *Lactobacillus* concentrations to compare to the growth in the presence of *Lactobacilli*. One hundred *μ*L were taken from the broth preparations before mixing with the *Lactobacilli* and were incubated along with the mixtures. The pH of the broth without *Lactobacilli* was taken after incubation to compare to the pH of that pathogen mixed with *Lactobacilli*. The growth of each pathogen used was inhibited by the *Lactobacilli*, and the broth pH was lowered. Utilizing these pathogens as controls proves that the conditions used in the methodology allowed the *Lactobacilli* to inhibit other organisms as they would in the body.

## 3. Results and Discussion

In each of the samples, no inhibition of *S. pyogenes* by *L. crispatus* and *L. jensenii* was observed. Statistical tests (*t*-tests with an alpha level of *α* = 0.01) showed no significant difference between the colony counts of *S. pyogenes* by itself and growth when mixed with *Lactobacilli*. In the case of most pathogens, a higher concentration of *Lactobacilli* would cause more inhibition. However, statistical tests (ANOVA, *α* = 0.01) also showed no significant difference between the colony counts of *S. pyogenes* in the four different concentrations of *Lactobacilli*. The difference in colony-forming units for each *S. pyogenes* isolate was most likely due to technical error during the serial dilutions and/or random error due to the differing strains of *S. pyogenes*.

The pH of the broth for each sample was reduced from the starting pH of 8.5 to a pH range of 4.5–7.0. The pH of the broth only containing *S. pyogenes* ranged from 5.5 to 6.5, suggesting that *S. pyogenes* grows optimally in an acidic environment [5]. Table 1 summarizes the average colony counts and pH of the mixtures of *S. pyogenes *and *Lactobacilli*.

 Limitations of this study included the elevated pH of the broth medium and technical error associated with serial dilutions. The process of serial dilutions allowed the possibility of pipetting error which may have influenced the colony counts. The elevated pH of the broth medium did not allow any data at a pH lower than 4.5. It is also possible that the alkaline pH of the broth medium impacted inhibition of the *Lactobacilli *and/or *S. pyogenes*. 

Additional studies that further investigate the prevalence and clinical significance of bacterial vaginosis caused by *S. pyogenes *would add to the current body of knowledge on this topic.

## 4. Conclusions

**Figure 1:**
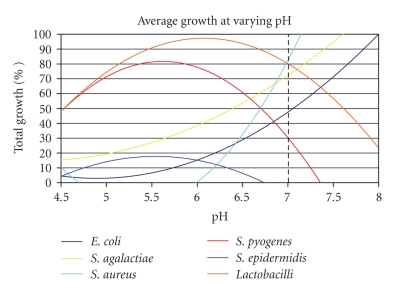
Percent of total growth of each organism comparing growth with *Lactobacilli* to growth without *Lactobacilli*. These data were taken from the results of this study.

**Figure 2:**
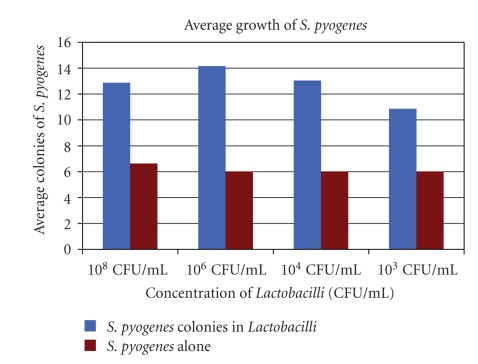
Average growth of *S. pyogenes*, suggesting that *Lactobacilli* may assist the growth of *S. pyogenes. *

Figure 1 shows the growth patterns of the organisms used in this study. With the exception of *S. pyogenes* and *S. epidermidis*, the pathogens grew optimally in conditions where the *Lactobacillus* concentrations were decreased. *S. epidermidis* is normal skin flora and is inhibited to nonpathogenic levels by the *Lactobacilli *within the vagina. *S. pyogenes* grew optimally in the same conditions as the *Lactobacilli*. It is possible that higher concentrations of *Lactobacilli *may assist the growth of *S. pyogenes*, as shown in Figure 2.

It is suggested that medical personnel treat vaginosis caused by *S. pyogenes* using antimicrobial therapy, as they would treat other *S. pyogenes* infections. It is also suggested that *Lactobacillus* probiotic therapy not be used as the sole means to treat bacterial vaginosis caused by *S. pyogenes*. The statistical data representing the growth of these two organisms suggest that *Lactobacilli* did not inhibit the growth of *S. pyogenes*. Also, *S. pyogenes* did not inhibit the growth of *Lactobacilli*.

## Figures and Tables

**Table 1:** Average growth and pH of the broth mixtures.

Colony counts from broth mixtures	10^8^ CFU/mL *Lactobacilli *	10^6^ CFU/mL *Lactobacilli *	10^4^ CFU/mL *Lactobacilli *	10^3^ CFU/mL *Lactobacilli *
Average colony counts of* Lactobacilli *	>100	>100	10	4
Average colony counts of* S. pyogenes *	13	14	13	11
Average pH	6.0	6.5	6.5	6.5
